# Histological Analysis of Biological Width and Collagen Fibers Orientation Around Screw-Less, Morse Taper, Hemispherical Base Abutments 8 and 16 Weeks After Implant Uncovering: An Observational Clinical Trial

**DOI:** 10.3390/dj13040154

**Published:** 2025-03-31

**Authors:** Fabrizio Zaccheo, Giulia Petroni, Marco Tallarico, Cherana Gioga, Raffaella Carletti, Cira Rosaria Tiziana Di Gioia, Vincenzo Petrozza, Silvio Mario Meloni, Dario Melodia, Milena Pisano, Andrea Cicconetti

**Affiliations:** 1 Department of Oral and Maxillo-Facial Sciences, Sapienza University of Rome, 00185 Rome, Italy; fabrizio.zaccheo@uniroma1.it (F.Z.); giulia.petroni@uniroma1.it (G.P.); andrea.cicconetti@uniroma1.it (A.C.); 2 Department of Medicine, Surgery and Pharmacy, University of Sassari, 07100 Sassari, Italy; mtallarico@uniss.it (M.T.); smeloni@uniss.it (S.M.M.); dariomld1@gmail.com (D.M.); 3 Faculty of Dentistry, Titu Maiorescu University, 1085 Bucharest, Romania; cherana.gioga@prof.utm.ro; 4 Department of Radiological, Oncological and Pathological Sciences, Sapienza University of Rome, 00185 Rome, Italy; raffaella.carletti@uniroma1.it (R.C.); cira.digioia@uniroma1.it (C.R.T.D.G.); 5 Department of Medical-Surgical Sciences and Biotechnology, University of Rome Sapienza, 04100 Latina, Italy; vincenzo.petrozza@uniroma1.it

**Keywords:** histological analysis, dental implants, healing abutment, biological width, SEM

## Abstract

**Objectives:** This study aimed to histologically evaluate, in humans, the orientation of collagen fibers around screw-less, Morse taper, hemispherical base abutments. **Methods:** This study was designed as an observational, case–control, clinical trial to evaluate the histological orientation of collagen fibers around implants. Biopsies of the peri-implant tissue were performed 8 (group A, control) or 16 (group B, test) weeks of implant uncovering, and histologically analyzed under optical microscope using Hematoxylin and Eosin, Masson, and Picro Sirius histochemical staining and a scanning electron microscope. **Results:** Eight patients were enrolled in this study and 16 biopsies were performed. All the biopsies were correctly analyzed. The histological examination of cross-sectional portions of the tissue taken 8 weeks after implant uncovering showed the almost complete absence of epithelial lining, while the connective tissue bundles in the superficial portion showed a lower circular pattern. The histochemical cross-section examination of the tissue taken 16 weeks after implant uncovering showed the partial presence of non-keratinizing epithelial lining at the implant site and the collagen bundles showed a greater organization, with a circumferential course around the abutment. At 8 weeks, the final histological analysis showed an average height of 1.01 mm for the keratinized epithelium, 0.83 mm for the non-keratinized epithelium, and 1.39 mm for the connective tissue. While, at 16 weeks, the values were 1.20 mm, 0.48 mm, and 1.11 mm, respectively. No statistically significant differences were found between the groups (*p* > 0.05). **Conclusions:** Histologically, there were not any differences in the height and profile of the gingiva between 8 and 16 weeks of healing after prosthesis delivery. Greater organization of the collagen fibers with a circumferential course around the abutment was found in the test group (16 weeks) compared with the control group (8 weeks).

## 1. Introduction

The success of an implant treatment is firstly determined by osseointegration, but is above all determined by maintaining the stability of both marginal bone and peri-implant soft tissues over time. Stable physiological marginal bone remodeling can be expected in the medium–long-term follow-up when using modern implant surfaces and connections [1]. In the last decade, the concept of biological width has been widely discussed in oral implantation. The characteristics of the peri-implant mucosa are determined during the biological width establishment that occurs following several weeks of healing after connecting the healing abutments or prosthetic components [2,3]. The biological width around an implant is a complex system of 3–4 mm in size. It extends from the top of the peri-implant mucosa to the first bone-to-implant contact, consisting of sulcular epithelium, junctional epithelium, and fibrous connective tissue [4,5]. However, the different arrangement of the fibers of connective tissue has been demonstrated around implants when compared with natural teeth, with circular or ring-shaped arrangements parallel to the long-axis and inserted implant. The role of the biological width is to create a biological barrier against the toxic products from bacteria, protecting the underlying bone [2]. In recent years, this concept has become very popular in modern dental implantology, which is more oriented towards the needs of soft tissue management. Soft tissue management can be carried out with different surgical techniques and at different times during implant treatments, based on the initial defect, the anatomical area, and aesthetic considerations. Briefly, keratinized tissue augmentation can be performed to obtain a band of keratinized tissue around implants that improves their aesthetic results and reduces the risk of plaque accumulation. On the contrary, soft tissue thickness augmentation can be performed to increase the thickness of the peri-implant supracrestal soft tissues that are crucial to obtaining a natural emergence profile for the prosthetic restoration and to ensuring a satisfying aesthetic result. Moreover, a thin biotype or phenotype often goes against resorption to restore the correct biological width [5,6]. Microgaps and micro-movements at the implant–abutment interface also play an important role in the stability of the peri-implant hard and soft tissues [7,8]. Micro-leakage at the implant–abutment interface may determine bacterial passage and the release of small titanium particles that induce inflammation in the peri-implant tissues [7]. For such reasons, today, implants with a Morse taper connection and without a connection screw may represent a good choice.

The supragingival fiber apparatus consists of a dense network of collagen fiber bundles that attach the gingiva to the teeth and bone while connecting the vestibular and oral interdental papillae [9]. This fiber network provides mechanical resistance, enabling the gingiva to withstand frictional forces from mastication. The junctional epithelium forms the coronal part of the dentogingival junction, while the apical portion consists of dentogingival connective tissue fibers extending into the root cementum, ensuring connective tissue attachment [9]. The peri-implant mucosa around successful endosseous implants shares many similarities with the tissues surrounding natural teeth [10]. The lamina propria extends coronally from the alveolar bone and is covered by keratinized oral epithelium. In healthy conditions, a shallow sulcus lined by sulcular epithelium forms. Among the tissues in contact with the implant, the junctional epithelium most closely resembles that of a natural tooth, forming a biological attachment via basal lamina and hemidesmosomes [11]. However, differences exist in how connective tissues interface with implants due to the absence of root cementum. While dentogingival fibers in natural teeth attach perpendicularly or obliquely to mineralized cementum, their direct attachment to implants remains debated. Most connective tissue fibers run parallel to the implant surface, either in a coronal–apical direction or circumferentially. Some studies suggest the presence of fibers oriented perpendicularly or obliquely to the implant surface, particularly on microtextured rather than smooth transmucosal surfaces [12,13].

The aim of this clinical trial was to preliminary evaluate, in humans, biological width measure, and the orientation of the collagen fibers around screw-less, Morse taper, hemispherical bases 8 and 16 weeks after implant uncovering. The following manuscript has been written according to the STROBE guidelines for reporting observational clinical trials.

## 2. Materials and Methods

This study was designed as an observational, case–control, clinical trial, and it aimed to assess the height and profile of the biological width as well as the orientation of collagen fibers around screw-less, Morse taper implant–abutment connections among participants at 8 and 16 weeks after implant uncovering. This study was conducted in accordance with the principles outlined in the Declaration of Helsinki for Biomedical Research Involving Human Subjects, as amended in 2018. The research protocol received ethical approval from the University of Bucharest in Romania (protocol number 15/2024, 16 December 2024) and was registered in an online database of clinical research studies (register number: NCT06862505; 5 March 2025). Any patient aged 18 years or older, affected by partial edentulism of posterior (premolars and molars) elements, needing at least two single-implant rehabilitations without the need for soft and/or tissue augmentation, and able to understand and sign an informed consent form, was considered eligible for this study. Patients were excluded if any of the following exclusion criteria were present: systemic or local contraindications for implant placement; untreated periodontal disease; smoking; osteoporosis; uncontrolled diabetes; the refusal of implant rehabilitation; the refusal of biopsy collection; and systemic pathologies that could compromise the healing of the peri-implant tissues. All the selected patients were informed about all the surgical and prosthetic procedures, including the benefits and the potential risks and complications of this research, and written informed consent was obtained before final enrollment.

### 2.1. Pre-Surgical and Surgical Protocol

Patients were initially subjected to clinical and instrumental investigations to find out their general health status and eligibility. The instrumental tests required by the department as part of the normal protocol were as follows: blood tests, ECG and cardiological examination, and Cone Beam CT of the dental arch. About 10 days prior to implant placement, all patients underwent professional oral hygiene sessions. All patients received prophylactic antibiotic therapy: 2 g of amoxicillin 1 h prior to the intervention or clindamycin 600 mg 1 h before implant placement if allergic to penicillin. All patients rinsed with chlorhexidine mouthwash 0.2% for 1 min prior to any surgical procedure. Local anesthesia (Septanest with adrenaline, 1/100,000, Septodont, Mataró, Spain) was administered with an infiltrative technique. The incision and elevation of a trapezoidal mucoperiosteal flap were performed at the chosen implant site. Once the bone was exposed, osteotomy was performed with a pilot guide of 2.0 mm diameter, at 1100 rpm, with external cold irrigation. The initial osteotomy was enlarged according to the manufacturer’s instructions. Short dental implants (Bicon system, Boston, MA, USA) for single- and multiple-tooth replacements were inserted 2 to 3 mm below the level of the marginal bone crest, and healing caps were used. The flap was repositioned and passively sutured to allow for primary intention healing. A two-staged surgical protocol was used. Three months after implant placement, local anesthesia (Septanest with adrenaline, 1/100,000, Septodont, Mataró, Spain) was performed with an infiltrative technique with a vasoconstrictor. All the implants were uncovered by making minimally invasive linear incisions on the ridge and removing all the growth tissues above. Once the healing cap was removed, a snap-on impression was taken. After that, a 4 (premolars) or 5 (molars) mm diameter polycarbonate healing abutment was inserted and engaged onto the implants. The patients were randomized into groups that received their definitive restorations 8 (control) or 16 (test) weeks after healing abutment connection in order to evaluate the healing of the peri-implant soft tissues over different time intervals. An intraoral radiographic examination of the implant site was conducted for all patients in order to evaluate the correct fixing of the healing abutment initially and their definitive restoration later. The Rinn alignment system was used.

### 2.2. Biopsy Collection Technique

**Figure 1:**
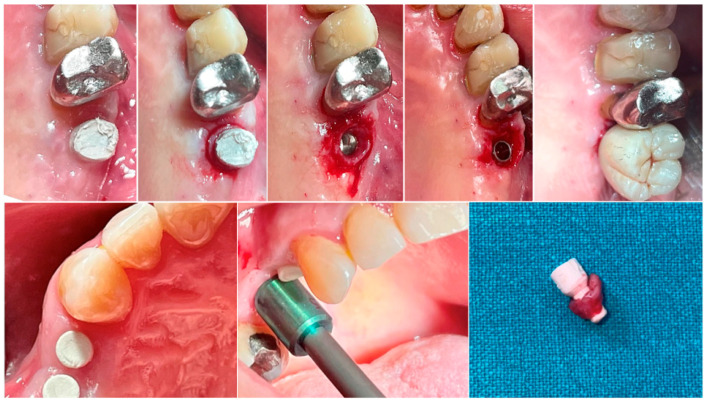
Biopsy sequence. Upper: Molar site. Lower: Premolar site.

In both groups, at different time intervals, a biopsy of the peri-implant soft tissue was harvested immediately before the delivery of the definitive prosthesis. Anesthesia was performed with an infiltrative technique 10 mm away from the sample site. A mucotome of 5 (premolars) or 6.5 (molars) mm in diameter was used around the hemispherical base abutments. Each sample included the soft tissues formed around the abutments during the healing period of 8 or 16 weeks (Figure 1). The samples were sectioned into two identical parts. The first collected tissue parts were fixed in 10% neutral buffered formalin for optical examination. The second parts were fixed in glutaraldehyde 2.5% in PBS 0.1 M pH 7.4 for at least 4 days for scanning electronic microscope observation. Post-surgical analgesic treatment with ibuprofen 600 mg was prescribed as needed. All the patients were strictly followed-up over the next six months to evaluate the complete healing of the harvested soft tissue.

### 2.3. Outcome Measures

The primary outcome was implant failure, defined as mobility, infection, fracture, and/or any other mechanical or biological issue that determined its removal. In addition, any biological (e.g., drug-resistant pain, swelling, excessive MBL, suppuration, etc.) and/or technical (e.g., fracture of the veneering material and/or framework, screw loosening, etc.) complications were recorded during follow-up.

The secondary outcomes were histological analysis with histomorphometry and scanning electron microscope (SEM) analysis.

All the histological samples were analyzed at the Department of Radiological, Oncological and Pathological Science, “La Sapienza”, University of Rome, Italy. For the histological diagnosis, two groups were defined: those who underwent 8 or 16 weeks of healing after implant uncovering. For histological analysis, the collected samples were further divided into two parts for both transversal and longitudinal examinations. All the samples were embedded in paraffin and cut using a microtome to obtain transversal and longitudinal sections to be used for the morphological analyses. For each formalin-fixed sample, two parts were embedded in paraffin wax, the first one cut along to the longitudinal axis to see the all long axes of the specimen, and the second one along the semi-circumferential axis to see the short axis of the specimen. The histological sections were stained with Hematoxylin and Eosin, Masson’s trichrome, and Picro Sirius, and observed by a Leica optical microscope (Leitz Camera, Wetzlar, Germany).

The organization of collagen fibers were evaluated through histological examination of the cross-sectional sections stained with Hematoxylin and Eosin, Masson’s trichrome, and Picro Sirius (with and without polarized light) and observed by a Leica optical microscope. Biological width was measured as the height and profile of the longitudinal histological sections analyzed with light microscopic analysis and histochemically stained with Hematoxylin and Eosin and observed by a Leica optical microscope. The organization of collagen fibers was also evaluated with a scanning electron microscope (ZEISS EVO 40, ZEISS, Oberkochen, Germany).

Quantitative, histomorphometric measures were evaluated on the longitudinal sections using ImageJ software 1.54 (National Institute of Health, Bethesda, MD, USA) at 1.6× magnification. Microscope standardization was ensured by using the same Leica optical microscope for the morphological analyses, and two expert researchers (R.C., C.D.G.), blinded to the type of sample, performed the evaluations and the histomorphological measures.

For the scanning electron microscope analysis, the samples underwent post-fixation in osmium tetroxide solution 2% in H_2_O for 2 h. The samples were then washed in H_2_O twice for 20 min in order to remove the post-fixation solution. Dehydration in an ascending series of alcohol solutions (30–50–70–95–100%) was then performed. In order to preserve their ultrastructural surface details, samples were dried in an Emitech K850, “critical point drying” apparatus (Emitech Ltd., Ashford, Kent, England). The dried samples were mounted by silver glue onto aluminum stubs and then sputter-coated with platinum (2 min, 15 mA) using an Emitech K 550 sputter coater (Emitech Ltd., Ashford, Kent, England). Samples were observed in high-vacuum conditions at 12 kV by a Hitachi SU3500 (Hitachi Ltd., Hitachi, Japan) scanning electron microscope.

### 2.4. Sample Size and Randomization

Sample size calculation was not performed due to the fact that present study was considered a pilot, observational clinical trial. A computer-generated randomization list was created. Only one of the investigators, not involved in the selection and treatment of the patients, was aware of the randomization sequence. The randomized codes were enclosed in sequentially numbered, identical, opaque, sealed envelopes. The envelopes were opened sequentially immediately after impression taking; therefore, treatment allocation was concealed to the investigators during the enrolling and treating of the patients.

### 2.5. Statistical Analysis

All analyses were carried out according to a pre-established analysis plan using SPSS software for Mac OS X (version 22.0; SPSS, Chicago, IL, USA). A dentist (M.T.) analyzed the data. Descriptive analysis was performed for numeric parameters using means ± SD. Differences in mean tissue compositions (height and profile) at 8 and 16 weeks were compared using paired *t*-tests. The patient was the statistical unit of the analyses. All the statistical comparisons were conducted with a 0.05 level of significance.

## 3. Results

**Figure 2:**
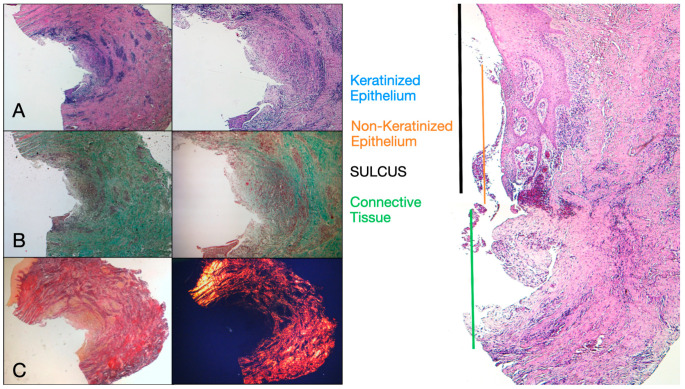
Histological cross-section of peri-implant tissue at 8 weeks after implant uncovering. (**A**) Histochemical staining with Hematoxylin and Eosin at 1.6× magnification (left) and 5× magnification (right). (**B**) Observation of histological sections stained with Masson’s trichrome observed at 1.6× (left) and 5× (right) under optical microscope. Type I collagen highlighted in green. (**C**) Histological sections stained with Picro Sirius observed at 1.6× under optical microscope; polarized light highlights type I collagen in red (right). Keratinized epithelium (blue), non-keratinized epithelium (orange), connective tissue (green), sulcus (black).

Eight patients aged between 38 and 63 years were selected, four women and four men (mean age 51.5 ± 9 years). Each patient received two biopsies at two different sites according to the randomization. At both 8 and 16 weeks after implant uncovering, clinical examination showed healthy peri-implant tissues. No patient dropped out. No implant failed during osseointegration and no biological or technical complications were experienced up to six months after prosthesis delivery. In the light microscopic analysis, the tissues showed a total absence of inflammation and an increased organization of collagen fibers arranged circumferentially to the abutment. The amount of tissue included in the samples allowed for the preparation of several sections with different colorings for both light and scanning electron microscopy. The histological examination of the cross-sectional sections taken 8 weeks after implant uncovering showed the almost complete absence of epithelial lining at the site of the implant, which was made up of loose connective tissue with small, newly formed vessels in the presence of focal hemorrhagic extravasations and lymphocytic inflammatory infiltrate. The adjacent connective tissue appeared denser, with abundant inflammatory infiltration consisting of lymphocytes and plasma cells predominantly arranged around small vascular structures. In addition, the connective tissue bundles in the superficial portion showed a circular pattern. During Masson’s trichrome and Picro Sirius examination, the latter, observed under an optical microscope with polarized light, demonstrated the almost exclusive presence of collagen type I (Figure 2).

**Figure 3:**
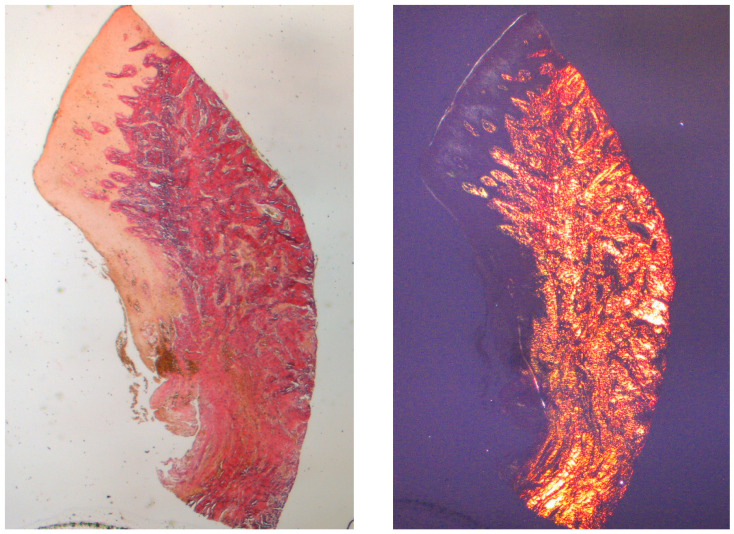
Longitudinal histological section at 8 weeks stained with Picro Sirius. Observed under light microscope at 1.6× magnification (**left**) and with polarized light (**right**).

The tissue analyzed in the longitudinal sections showed how the sulcus area is divided into two parts, characterized by an area starting from the gingival margin lined by keratinizing Malpighian epithelium and an area lined by epithelium with less evident keratinization. The latter is continuous with a de-epithelialized area consisting exclusively of loose connective tissue with newly formed vessels. In the context of the connective tissue, there is a lymphocytic and plasma cell inflammatory infiltrate in the sub-epithelial and perivascular areas (Figure 1). The Picro Sirius staining of the longitudinal section taken 8 weeks after implant uncovering shows the presence of some bundles of collagen, originating from the portion of connective tissue adjacent to the bone, which run vertically towards the free margin of the gingiva, along with a substantial disorganization of the other collagen fibers (Figure 3).

**Figure 4:**
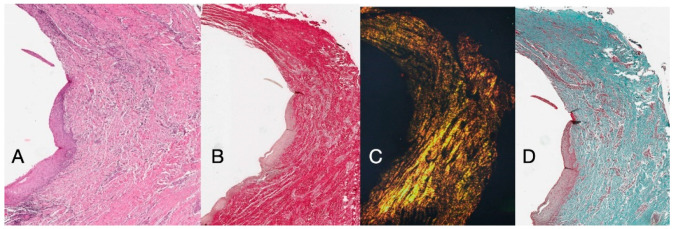
(**A**) Histological cross-section portion of peri-implant tissue at 16 weeks after implant uncovering. Histochemical staining with Hematoxylin and Eosin. Magnification 16×. (**B**) Histological section stained with Picro Sirius observed at 1.6× magnification; (**C**) 1.6× magnification Picro Sirius-stained sample observed with polarized light. (**D**) Histological section stained with Masson’s trichrome at 1.6×. Type I collagen colored green.

Histochemical cross-section examination of the tissue taken 16 weeks after implant uncovering shows the partial presence of non-keratinized epithelial lining at the implant site, which is made up of mature loose connective tissue free of inflammatory infiltrate. The outermost connective tissue is more organized than in the previous sampling. The collagen bundles showed a greater organization with a circumferential course around the abutment. Staining with Masson’s trichrome and Picro Sirius, the latter also observed with polarized light, demonstrated the almost exclusive presence of type I collagen. The images obtained with these stains show more clearly the organization and orientation of the circular fibers. Staining of the longitudinal section with Picro Sirius shows, despite the artifacts, the vertical organization of the collagen fibers in the lower portion of the piece (Figure 4).

**Figure 5:**
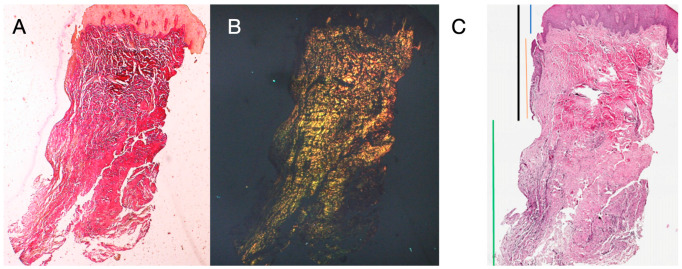
Longitudinal histological section stained with Picro Sirius. Observed under light microscope at 1.6× magnification (**A**), and with polarized light (**B**). Measurements of height and profile of samples at 16 weeks after implant uncovering according to different tissues (**C**). Keratinized epithelium (blue), non-keratinized epithelium (red), connective tissue (green), sulcus (black).

The histochemical longitudinal sections taken 16 weeks after implant uncovering showed sulcus areas divided into an area starting from the gingival margin lined with keratinizing Malpighian epithelium and an area lined with non-keratinized epithelium. The latter is continuous with a de-epithelialized area consisting exclusively of connective tissue. The connective tissue was shown to be composed of type I collagen and free of inflammatory infiltrate (Figure 5).

Finally, histological analysis showed no statistically significant differences between soft tissue composition at both time intervals for both height and profile. The mean biological widths measured at 8 and 16 weeks were 3.2 and 3.1 mm, in height, and 5.5 in profile at both follow-ups (Table 1).

**Figure 6:**
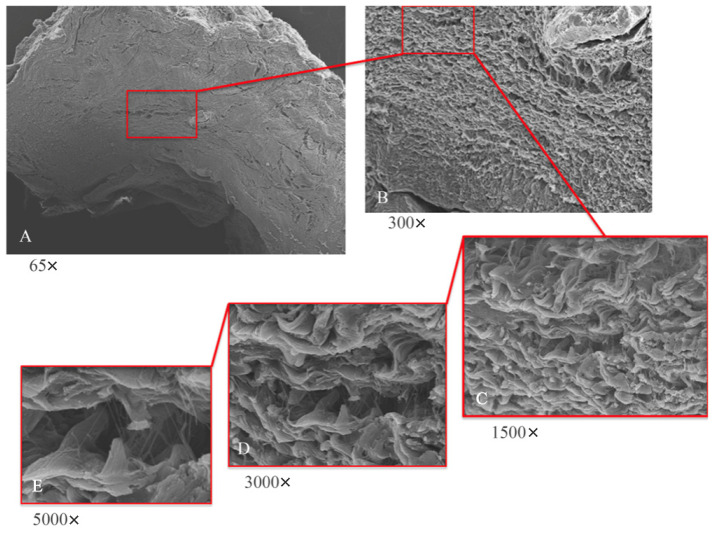
SEM analyses at lower (**A**,**B**) and higher magnifications (**C**–**E**). (**A**) SEM; panoramic view of implant abutment (65×); tissue shows absence of inflammation. (**B**) SEM; typical mature connective tissue; rich in regularly arranged collagen fibers with main circular orientation surrounding abutment (300×). (**C**) SEM; wavy collagen laminae arranged in parallel and orderly manner (1500×). (**D**) SEM; visible areas rich in thin collagen fibers projecting from collagen laminae (3000×). (**E**) SEM; thin collagen fibers arise from laminae as single fibers; they do not collect into bundles.

Low-magnification electron microscope observation (Figure 6A) of the samples shows the peri-implant mucosa to be free of inflammation, edema, or blood extravasation. Around the healing abutment, the presence of mature connective tissue is observed, rich in orderly organized collagen fibers. At higher magnifications (Figure 6B,C) wavy collagen fibers are visible, organized in an orderly and parallel manner; they have a predominantly circular organization around the abutment.

To analyze this dense matrix in more detail, images at 300× and 500× magnification were captured with a scanning electron microscope. At these magnifications, fine collagen fibers can easily be observed that arise from the basement membrane (Figure 6D,E) and that project as single fibers not organized in bundles.

## 4. Discussion

Today, dental implants are a common and extremely effective procedure to restore missing teeth [14]. Investigation into the factors that keep peri-implant tissues in a stable and long-term healthy state has been the main focus of research on implants [15]. To reduce marginal bone loss and maintain peri-implant soft tissue levels, different approaches have been established, including micro- and macro-implant designs, surgical and prosthodontics procedures, platform switching, and implant–abutment connections [15,16,17].

The primary function of the periodontal tissues, besides attaching the tooth to the jaw, is gingival protection; that is, their function is to provide a seal against the contaminated environment of the oral cavity, to withstand the frictional forces of mastication, and to defend the interface between the teeth and soft tissue against foreign invaders. The biological width around the implant is different from that around natural teeth in many ways, including concept, formation, remodeling, size, and structure, and has an important role in the remodeling of peri-implant soft and hard tissues. In the present study, peri-implant soft tissue harvests were performed at 8 and 16 weeks after implant uncovering and the placement of a polycarbonate hemispherical healing abutment. Eight weeks of healing was chosen as the control condition. The presence of a natural barrier of connective tissue and epithelium at 8 weeks is in agreement with several studies that conducted animal experiments [18,19,20]. However, the harvests taken at 8 weeks showed modest remodeling, with perivascular lymphocytic and plasma cell infiltrate and the peri-implant tissues having morphostructural characteristics superimposed on healthy tissues. Samples at 16 weeks showed a total absence of inflammation and an increased organization of collagen fibers arranged circumferentially to the abutment. Furthermore, in the longitudinal histological sections there was a morphostructural pattern where the sulcus area was divided into two parts, with one area starting from the gingival margin lined by keratinizing Malpighian epithelium and one area lined by epithelium with less obvious keratinization (JE). The main arrangement of the fibers was parallel in the longitudinal cuts (Figure 4), with respect to the implant axis, and circular in the transverse sections (Figure 3). At higher magnifications, 300× and 500× (Figure 6B,C) wavy collagen fibers arranged in an orderly and parallel manner could be observed. These fibers primarily exhibited a circular organization around the abutment. Fine collagen fibers arising from the basement membrane and projecting as single fibers not organized in bundles could be easily observed in the 3000× and 5000× SEM images (Figure 6D,E).

The present study showed that a hemispherical abutment design facilitates the formation of a structured connective tissue cuff with highly aligned fibers, as compared to the random distribution seen in parallel-walled abutments. This suggests that ECM organization can be influenced by macro-scale tissue geometry, guiding fiber arrangement, and morphogenesis [21]. Similar studies have described this effect in platform-switching implants, where collagen orientation provides mechanical retention for periodontal fibers [21,22,23]. Animal models have also reported supracrestal circular collagen fiber networks comparable to gingival ligaments [5,24].

A recent preclinical study [25] demonstrated that a concave transmucosal design could promote greater connective tissue deposition and growth compared to a straight design. Findings indicated an increase in connective tissue thickness, a denser peri-implant network, and collagen fiber alignment toward the abutment collar, forming a broad circular collagen structure around the implant platform. The present study supports these observations, as the introduction of a hemispherical profile facilitated the arrangement of collagen fibers into well-organized parallel bundles.

Collagen fiber orientation is a key biomechanical factor, reflecting the forces acting upon the connective tissue, particularly through collagen bundles. Research has shown that an excessive accumulation of randomly oriented collagen can lead to dysfunctional fibrotic tissue formation [26]. The directional organization of collagen fibers is crucial for peri-implant soft tissue stability, as noted by Karjalainen et al. [27].

In the present study, the mean biological widths were 3.2 and 3.1 mm at 8 and 16 weeks, respectively. Comparing the present results with other similar studies, in a study by Tommasi et al. [28], at 8 weeks after uncovering the average value was 2.7 mm, including 1.5 mm of epithelium and 1.2 mm of connective tissue, while the profile dimension was 3.6 mm. In the present study, the mean biological widths were a little bit higher in both epithelium and connective tissue. However, a great increase was found for the overall profile. The main difference is that, in the Tommasi study, the second follow-up was at 12 weeks instead of 16 weeks. A possible explanation for this difference is that tissues continue to grow for at least 4 months after reopening.

Through SEM it is also possible to find wavy collagen fibers organized in an orderly and parallel manner, with a predominantly circular organization around the abutment. In relation to this, it can be assumed that the predominant organizations of the circular collagen bands and the vertical collagen bands were organized in relation to the abutment design. From this point of view, the possibility of using implants or implant–crown systems that favor the stability of the mucous seal represents an important area of research [17]. This solution shifts the connective seal apically in relation to the prosthetic components, whose micro-movements—often preventing stable connective tissue formation—without compromise the final stabilization of the seal. The main limitation of the present research was the small sample size, due to the fact that a sample size calculation was not performed. Further clinical trials with larger samples are needed in order to confirm these preliminary results.

## 5. Conclusions

Histologically, there were not any differences in the height and profile of the gingiva between 8 and 16 weeks of healing after implant uncovering. At 16 weeks, more tissue was found in both groups. A greater organization of the collagen fibers into circumferential courses around the abutments were found in the test group (16 weeks) compared with the control group (8 weeks).

## Figures and Tables

**Table 1:** Soft tissue composition at in both time intervals.

	HEIGHT					
	Group A—8 weeks			Group B—16 weeks		
	Keratinized Epithelium	Non-Keratinized Epithelium	Connective Tissue	Keratinized Epithelium	Non-Keratinized Epithelium	Connective Tissue
Mean	1.01 mm	0.83 mm	1.39 mm	1.20 mm	0.84 mm	1.11 mm
*p* Value	0.057	0.948	0.165			
	**PROFILE**					
	Group A—8 weeks			Group B—16 weeks		
	Keratinized Epithelium	Non-Keratinized Epithelium	Connective Tissue	Keratinized Epithelium	Non-Keratinized Epithelium	Connective Tissue
Mean	1.37 mm	1.92 mm	2.24 mm	1.27 mm	1.86 mm	2.4 mm
*p* Value	0.089	0.491	0.431			

## Data Availability

No new data were created or analyzed in this study.
